# Petri Net modeling of thiamine diphosphate biosynthesis in *Mycobacterium tuberculosis* H37Rv

**DOI:** 10.6026/973206300211029

**Published:** 2025-05-31

**Authors:** Mohd Asif Siddiqui, Ravindra Kumar Jain, Ashwani Kumar, Dhanendra Kumar Rai, Nikhil Chand, Ritika Yadav, Anu Chauhan, Sarita Rana

**Affiliations:** 1 Department of Biotechnology, Swami Vivekanand Subharti University, Subhartipuram, NH-58, Delhi-Haridwar Bypass Road, Meerut-250005, India

**Keywords:** Thiamine biosynthesis, Petri nets, modelling, simulation, *Mycobacterium tuberculosis*

## Abstract

Thiamine diphosphate (TPP) is essential cofactor in *Mycobacterium tuberculosis* H37Rv metabolism, making its biosynthesis pathway a key target for therapy. Therefore, it is of interest to describe a Petri net-based model of the TPP biosynthesis super-pathway, developed using curated MetaCyc data and simulated with Snoopy software. The model integrates three biosynthetic branches and maps key enzymes (ThiC, ThiD, ThiE, ThiF, ThiG, ThiS) along with their gene identifiers. The simulation of token flow revealed the pathway's dynamics, highlighting critical regulatory nodes. This computational approach provides insights into TPP biosynthesis and serves as a basis for drug design targeting tuberculosis.

## Background:

*Mycobacterium tuberculosis*, the causative agent of tuberculosis, remains a major global health concern, responsible for over 1.5 million deaths annually [1]. Its slow metabolic rate contributes to drug resistance, complicating treatment efforts [2]. Characterizing its metabolic pathways is important for the development of new therapies. Among these, thiamine diphosphate (ThDP) biosynthesis is vital for the bacterium's survival, as thiamine functions as an essential metabolic cofactor [3]. Disrupting ThDP synthesis or function could impair bacterial metabolism, enhancing antibiotic efficacy. Petri nets - a dynamic modeling tool - effectively simulate such pathways by representing metabolites and reactions as places and transitions, capturing system behavior, regulation and interactions [4, 5]. Thus, modeling ThDP biosynthesis in *M. tuberculosis* using Petri nets provides insights into potential drug targets. Computational biology, an interdisciplinary field integrating computer science, mathematics and biology, offers effective tools to analyze the complexity of living systems [6]. Among these, Petri nets stand out as a graphical and mathematical framework for modeling dynamic biological processes.

Developed by Carl Adam Petri in the 1960s, Petri nets effectively represent concurrent, asynchronous and distributed systems, making them ideal for simulating biochemical pathways, gene regulation and cellular networks. A Petri net consists of places (states), transitions (events), tokens (conditions) and arcs (flow), enabling formal and intuitive modeling of system behavior [7, 8]. By simulating these networks, researchers can analyze system dynamics, predict responses to perturbations and identify critical control points [9]. Their graph-theoretic foundation further facilitates structural analysis and discovery of underlying biological principles [10]. Therefore, it is of interest to describe the development of a Petri net-based model of thiamine diphosphate biosynthesis I in *Mycobacterium tuberculosis* H37Rv. This model provides insights into the pathway's kinetics, regulatory mechanisms and its potential as a therapeutic target for tuberculosis.

## Metabolic networks:

Mathematical modeling of metabolic networks helps integrate complex biological data into a unified, consistent framework, enabling logical analysis of system components and interactions [5]. These models support simulation, prediction and optimization of experimental designs and therapeutic strategies, while enhancing our understanding of key system properties [11]. In addition to Petri nets, other modeling approaches - such as ordinary differential equations (ODEs), Markov chains, Bayesian networks and stochastic models - are widely used across various biological domains [12, 13]. A Petri Net (PN) models a metabolic pathway by representing biological entities-such as metabolites, enzymes and compounds-as places and chemical reactions as transitions [14]. Input places denote substrates, while output places represent reaction products. Arc weights reflect stoichiometric coefficients, capturing the quantitative relationship between reactants and products. Tokens indicate the quantity of each component at a given time and transition rates follow kinetic laws governing reaction speed and probability within the network.

## Methodology:

Data for the Super pathway of Thiamine Diphosphate Biosynthesis I in *Mycobacterium tuberculosis* H37Rv was sourced from the MetaCyc database - a curated repository of metabolic pathways and enzymes [15]. Key information, including reaction names, reactants and products, Enzyme Commission (EC) numbers, enzyme names and synonyms, was systematically extracted. Using this curated data, a Petri net model was constructed with Snoopy, a graphical tool for modeling and analyzing complex biochemical networks [16]. Snoopy enables detailed visualization and offers analytical modules for examining system dynamics and computing performance metrics.

## Results:

Thiamine diphosphate (TPP) biosynthesis I in *Mycobacterium tuberculosis* H37Rv is a complex, multi-step pathway involving three major biosynthetic routes (Table 1). It synthesizes two moieties - pyrimidine (HMP-PP) and thiazole (HET-P) - through distinct branches, which are then condensed to form thiamine monophosphate (TMP), subsequently phosphorylated into the active coenzyme TPP. Notably, this pathway is absent in humans, making its components attractive targets for anti-tuberculosis drug development (Figure 1 see PDF). The pyrimidine branch begins with the conversion of 5-aminoimidazole ribonucleotide (AIR) to HMP-P by phosphomethylpyrimidine synthase (ThiC, Rv0414c), followed by phosphorylation by HMP-P kinase (ThiD, Rv0415c) to yield HMP-PP. Concurrently, the thiazole moiety is synthesized from L-cysteine and glycine through the coordinated action of ThiS (Rv0423c), ThiF (Rv0424c) and ThiG (Rv0425c), forming HET-P. These intermediates are joined by thiamine phosphate synthase (ThiE, Rv0426c) to generate TMP, which is further converted to TPP, by a putative thiamine pyrophosphokinase (TPK1, EC 2.7.6.2). The gene encoding TPK1 remains unannotated, representing a gap in genomic data. Comprehensive details on enzyme functions, reaction equations, gene identifiers and compound abbreviations with their full names are presented in Table 2, Table 3 and Table 4. To understand pathway dynamics, a petri net model was constructed and simulated to analyze token flow, representing the state of each metabolite (Figure 2 see PDF). Simulations revealed enzymes such as ThiC and ThiG as key control points; their disruption halts TPP synthesis, underscoring their non-redundant and essential roles. Gene-enzyme mapping confirmed the specific roles of each component, with no known human homologs. The two-branch system converging on a single essential coenzyme emphasizes the need for coordinated regulation. The unannotated TPK1 step requires further investigation to confirm its genetic basis and druggability. In summary, the TPP biosynthesis I pathway in *M. tuberculosis* is indispensable for bacterial metabolism and absent in humans. Its enzymes, particularly those in the early steps of each branch, offer strong drug target potential. Integration of biochemical data with computational modeling provides a solid foundation for advancing anti-TB therapeutic strategies.

## Conclusion:

The value of PNs in modeling the thiamine diphosphate biosynthesis I pathway in *Mycobacterium tuberculosis* H37Rv, both quantitatively and qualitatively is shown. The study clarifies the pathway's structure and offers insights into key regulatory mechanisms. Modeling this pathway using Petri nets offers a potential approach for targeting thiamine diphosphate biosynthesis I as a strategy to combat tuberculosis. By simulating the dynamics of thiamine diphosphate biosynthesis, Petri nets enhance our understanding of its regulation and open new possibilities for drug development.

## Figures and Tables

**Table 1:** Key components: thiamine diphosphate biosynthesis I

**Route**	**Product**	**Precursor**
Ribose Pathway	HMP-PP (Pyrimidine moiety)	5-Aminoimidazole ribonucleotide
Thiazole Pathway	HET-P (Thiazole moiety)	L-cysteine, glycine, NAD^+^
Final Condensation	Thiamine monophosphate (TMP) → TPP	HMP-PP + HET-P + ATP

**Table 2:** Enzymes and reaction equations

**Pyrimidine Moiety (HMP-PP) Biosynthesis**			
**Step**	**Enzyme**	**EC Number**	**Reaction**
1	Phosphomethylpyrimidine synthase (ThiC)	EC 4.1.99.17	5-Aminoimidazole ribonucleotide (AIR) → 4-amino-5-hydroxymethyl-2-methylpyrimidine phosphate (HMP-P)
2	Phosphomethylpyrimidine kinase (ThiD)	EC 2.7.1.49	HMP-P + ATP → HMP-PP
**Thiazole Moiety (HET-P) Biosynthesis**			
1	Cysteine desulfurase (IscS / ThiI)	EC 2.8.1.7	L-cysteine → S-bound sulfur (for ThiS)
2	ThiF	EC 6.3.2.16	Activates ThiS via adenylation (ATP-dependent)
3	ThiS (sulfur carrier protein)	-	Carries sulfur group
4	ThiG (Thiazole synthase)	EC 2.8.1.10	Catalyzes thiazole ring formation
5	ThiO (glycine oxidase or oxidoreductase)	EC 1.4.3.19 (or equivalent)	Glycine → glycine imine → 5-hydroxyethyl intermediate
6	Thiazole phosphate synthase	EC 2.8.1.10	Forms THZ-P (4-methyl-5-(β-hydroxyethyl)thiazole phosphate)
**Thiamine Monophosphate (TMP) Formation**			
1	ThiE (Thiamine phosphate synthase)	EC 2.5.1.3	HMP-PP + THZ-P → Thiamine monophosphate (TMP)
**Conversion to TPP (Active Form)**			
1	Thiamine pyrophosphokinase (TPK1)	EC 2.7.6.2	TMP + ATP → Thiamine diphosphate (TPP)

**Table 3:** Gene identification (Gene ID) and enzyme names along with their functions

**Enzyme**	**Gene ID**	**Function**
ThiC	Rv0414c	HMP-P synthesis
ThiD	Rv0415c	HMP-P → HMP-PP
ThiS	Rv0423c	Sulfur transfer
ThiF	Rv0424c	ThiS activation
ThiG	Rv0425c	Thiazole ring formation
ThiE	Rv0426c	TMP synthesis
TPK1 (putative)	- (Not clearly annotated)	TMP → TPP

**Table 4:** Abbreviations of compounds and their full name

**S. No.**	**Abbreviation**	**Full Name**
1	AIR	5-Aminoimidazole Ribonucleotide
2	HMP-P	4-Amino-2-methyl-5-hydroxymethylpyrimidine Phosphate
3	HMP-PP	4-Amino-2-methyl-5-hydroxymethylpyrimidine Diphosphate
4	THZ-P / HET-P	4-Methyl-5-(β-hydroxyethyl)thiazole Phosphate
5	TMP	Thiamine Monophosphate
6	TPP	Thiamine Diphosphate
7	CTP	Cytidine Triphosphate
8	CMP	Cytidine Monophosphate
9	ATP	Adenosine Triphosphate
10	ADP	Adenosine Diphosphate
11	CO_2_	Carbon Dioxide
12	H^+^	Proton
13	EC	Enzyme Commission Number
14	NAD^+^	Nicotinamide Adenine Dinucleotide (oxidized form)
15	L-cysteine	L-Cysteine (used as a sulfur donor in thiazole biosynthesis)
16	Glycine	Glycine (provides the carbon backbone for thiazole synthesis)
